# Bury Me in the Churchyard at Home

**Published:** 1887-04-16

**Authors:** 


### "BURY ME IN THE CHURCHYARD AT HOME."

### A TRUE STORY OF SIMPLE HEROISM.

In the hospital ward she is lying,
With the hectic flush on her cheek,
And so near her end that her voice is faint,
And you scarce can hear her speak.

And sitting by the bedside
Of that girl so pale and wan,
With her thin white hand in his horny one,
Is a plain old labouring man.

When she was an orphaned baby,
To his village home he had brought her,
With pity and love encompassed round,
She had been to him as a daughter.

And when he was told she was failing—
That her hope of life was o'er,
He had trudged to the city full fifty miles,
To see her face once more.

"I have done so little for thee,
So little—but 'twas my best,
Now the Father in heaven will take thee home,
And give thee peace and rest.

"But what is it thou wouldst ask me,
My darling, tell me true—
For I see a longing in thine eyes,
What is it that I can do ?"

"Oh ! let me not here be buried,
Where none but strangers pass,
Oh ! take me home to our old churchyard—
Lay me under the daisied grass."

And his deep voice answered slowly,
"My darling, have no fear,
When the time is come, I will take thee home,
I will not leave thee here."

She knew she could trust his promise,
And that her fears might cease ;
That very night she passed away
With a smile of perfect peace.

The father took her coffin,
With tearless eyes, but sad,
And laid it on a barrow—
The only bier he had.

"You have done your best for my darling,
Your kindness will God repay,"—
So he spake, and wheeled his burden
From the hospital gates away.

Through London street and suburb,
Through country road and lane,
By day and night full fifty miles,
Till he reached his home again.

He kept his word to his darling,
And under the daisied grass
She was laid to rest in the old churchyard,
Where no strange footsteps pass.

Then, looking for no man's praises,
When his task of love was o'er,
The father went back to his daily toil,
And wrought for his bread once more.

